# Diseases of the Ear

**Published:** 1879-11

**Authors:** S. J. Jones

**Affiliations:** Illinois Charitable Eye and Ear Infirmary


CHICAGO MEDICAL

JOURNAL & EXAMINER.

VOL. XXXIX.—NOVEMBER, 1879.—NO. 5.

[In these pages dimension and weight are expressed in terms of the Metric System, and temperature in degrees of the Centigrade Scale.]

### Original Lectures.

ARTICLE I.

CLINICAL LECTURE. By PROF. S. J. JONES, at the Illinois Charitable Eye and Ear Infirmary. Reported by F. C. Schaefer, M.D., Assistant Aural Surgeon.

The diseases of the ear are classified in three divisions, corresponding to the anatomical arrangement of the organ viz:

I. Diseases of the external ear.
II. Diseases of the middle ear.
III. Diseases of the internal ear.

In order that you may fully comprehend the limits of each division, it will be well to review briefly the anatomy of the parts. The external ear comprises the auricle and external meatus to the membrana tympani. The middle ear or tympanum is an irregular cavity placed between the external and the internal ear, within the petrous portion of the temporal bone, having four irregular walls, a roof and a floor, and lined by a mucous membrane. It communicates with the pharynx through the eustachian tube. This cavity is separated from the external ear by the membrana tympani. A chain of bones, three in number, the ossicula auditus, within the middle ear, connect the membrana tympani with its internal wall, which lies in juxtaposition with the internal ear. The internal ear or labyrinth, placed next to the tympanum and separated from the latter by a thin partition wall of osseous and membranous tissue, is also an irregular cavity and contains the vestibule, semi-circular canals, cochlea and auditory nerve.

The external and middle ears, together, are recognized functionally, as conducting apparatus; the internal ear as the perceptive apparatus.

Your attention will be directed to the pathological conditions of the ear in the order named, according to the classification of Roosa.

Diseases of the external ear are:

(a.) Diseases of the auricle.
- I. Malformations.
- II. Tumors.
- III. Malignant growths.
- IV. Injuries.
- V. Eczema.

(b.) Diseases of the auditory canal.
- I. Malformations.
- II. Diffuse inflammation.
- III. Circumscribed inflammation.
- IV. Vegetable fungous growths.
- V. Inspissated cerumen.
- VI. Eczema.
- VII. Foreign bodies.
- VIII. Polypi.
- IX. Exostoses and hyperostoses.
- X. Syphilitic condylomata and ulcers.

The diseases of the middle ear are:

I. Acute catarrhal inflammation.
II. Sub-acute catarrhal inflammation.
III. Chronic non-suppurative inflammation—catarrhal or proliferous.
IV. Acute supurative inflammation.
V. Chronic suppurative inflammation.
VI. The consequences of chronic suppurative inflammation.

1. Polypi.
2. Exostoses.
3. Mastoid disease.
4. Caries and necrosis.
5. Cerebral abscess.
6. Pyæmia.
7. Paralysis.

The diseases of the internal ear manifest themselves as nervous deafness, and are classed according to their causes.

*Proximate Causes.*

Injuries.
Hæmorrhage and effusions.
Inflammation of the membranous (labyrinth.)
External administration of quinine (?)
Concussion of the nerve and its expansion.

*Remote Causes.*

Syphilis.
Cerebro spinal meningitis.
Fevers.
The exanthemata.
Mumps.
Cerebral tumors.
Aneurism of the basilar artery.

*Circumscribed Inflammation of the External Auditory Meatus.*

This patient, aged 24, complains of defective hearing; says he has great pain and a "lump" in his right ear, and that the hearing became "dull" during the past week. He says his hearing never has been impaired before. There is no tinnitus aurium. The borders of the external meatus are very tender, pressure upon the tragus increases the pain. Upon examination with the speculum, a circumscribed swelling or furuncle is to be seen upon the posterior wall of the external auditory canal near the entrance; it is very red, so large as to almost close the canal. Frequently several of these swellings appear at the same time, or as soon as one disappears another occurs. There being no tinnitus aurium, it is not probable that there is any lesion in the tympanum. As the furuncle is very tender, it will be advisable to scarify it through the periosteum, thereby reduce the congestion, diminish the tension upon the tissues and relieve the pain. When these furuncles are small, they can sometimes be aborted by making them bleed freely, and the hardened base that remains will be removed by absorption. When however the inflammation has continued for some time and the swelling is a little "boggy," the better plan is to encourage suppuration by lubricating the meatus with vaseline, and filling the ear with tepid water, and allowing it to remain for several hours.

As soon as it softens, puncture the swelling down to the bone. The after-treatment will consist in cleansing the parts and lubricating with vaseline. Should the pain continue, the warm water may be reapplied to the meatus and kept there with a cotton plug. The continuous application of warm water has a most felicitous effect in relieving pain within the ear.

Incising the furuncle gives immediate relief. Considerable blood escapes. This will relieve the tension of the parts, and if pus should yet form, it will have a more ready exit. Occasionally granulations occur. Should any appear, later, in this case, they will be touched with nitrate of silver, but caustics should be used with care in the external meatus, for excessive use of them sometimes causes these boils to form, and as they are inflammation of ceruminous glands, disease of one gland is liable to affect the nutrition of others, and thus produce a succession of boils, just as occurs in styes in the eye-lids, and in inflammation of axillary glands. Especially is this likely to occur in atonic conditions of general system.

*Inspissated Cerumen in the External Auditory Canal.*

This patient may be regarded as a typical case of a very large class. She states that she became suddenly deaf in the left ear; that she has ringing noises in the ear, a feeling of fullness about the left side of the head, and is frequently dizzy. The patient uses the inaccurate term that not only the laity but also some physicians employ when speaking of impaired hearing, and which it would be well for you to guard against. Notice when the watch is placed close to the ear she hears it; surely then she is not deaf, her hearing is defective; it has become impaired, not destroyed.

In the attempt to arrive at a correct diagnosis it is always best to make the examination systematically. Having ascertained that the patient's hearing is defective, the next step will be to examine the ear and see what objective symptoms are present. Introducing the otoscope in the external meatus, it meets with resistance ; this at once indicates either an unusual formation of the parts or the presence of a foreign substance in the canal. Looking through the otoscope there can be seen a dry, very dark substance, mixed with bits of hair and bran-like granules—dry epithelial scales. By probing this accumulation gently with a delicate scoop, a slightly grating sound is elicited, showing that it is quite hard ; a little pressure against the impaction produces roaring noises in the ear, together with pain and vertigo. This leads to the conclusion that the plug lies in intimate contact with the membrana tympani and fills the entire canal. The meatus is very dry, and about its borders there is an accumulation of dry epithelium, resulting from the dryness of the derma, occasioned by the suppression of the secretions. This mass consists of inspissated cerumen. A very plain case, easily diagnosed, it may seem ; and yet simple as it is to make a diagnosis, it may surprise you to be informed that physicians have frequently mistaken such cases for inflammation of the tympanum. There is however no excuse for this mistake. It simply argues negligence on the part of the doctor who in all probability attributed the fullness of the head and defective hearing to inflammation of the middle ear, without making an examination of the external meatus, and perhaps advised the patient to drop lotions or oils of various kinds into the ear, which tended to obscure the case and aggravate the symptoms. The suddenness of the impairment of hearing may be attributable to having gotten a little water into the meatus, in bathing, which was absorbed by the mass, and made it swell and more effectually close the passage.

The *etiology* is sometimes obsure. Among the most common causes of such accumulation are chronic non-suppurative inflammation of the middle ear and inflammation of the meatus — due to irritation produced with scoops, hair pins or other substances used to " clean " the ear, which is unnecessary in a healthy condition of the parts. The inflamed state of the skin occasions a dryness of its surface by suppression of its secretions as in fevers. This is accompanied by the evaporation of the liquid portion of the cerumen, leaving the dry residue in the canal, which in turn acts as an irritant and keeps the parts abnormally dry. It may accumulate in consequence of wiping the meatus with the end of a towel or the finger, thus forcing the wax back into the canal as it is secreted, compressing it. A slight swelling of the canal is liable to give rise to it by preventing the free removal of the cerumen which normally takes place from the motion of lower jaw.

These are among the causes that produce impaction. The largest number of cases arise from the extension of an inflammation of the middle ear to the inner portion of the external meatus. The records of this institution for the past year show that over 80 per cent. of these cases were accompanied by chronic non-suppurative inflammation of the tympanum. It becomes important, therefore, after removing the mass, to see if there is a lesion of the middle ear.

*Treatment.*—The removal of this dry hard substance is not always an easy task, and the utmost gentleness is called for in the effort. The meatus is to be thoroughly syringed with warm water containing bi-carbonate of soda, alkalies being the best solvent of ear-wax. The pinna is to be drawn upwards, outwards and backwards to straighten the canal, then the water is to be injected in a continuous stream from a large syringe This produces a steady pressure, and will aid materially in its removal. Any that may remain after syringing may be removed by an ear-scoop, gently manipulated. In the greater number of cases this treatment will suffice. However, occasionally cases are met with in which the impaction is such as to require no small degree of care to effect its removal without doing violence to the meatus, which would result in circumscribed or diffuse inflammation of it. This mass of ear-wax is sometimes found encased in a pocket of desquamated skin—a cast of the meatus—that by its hardness and pressure has caused absorption around it and enlarged the meatus.

After removal of such a mass it is well to keep the meatus lubricated, as with vaseline (many of the oils popularly used to soften wax and lubricate the ear are supposed to give rise to the vegetable parasite in the ear called aspergillus) thus to protect it until wax shall again be normally secreted.

Morbid sensitiveness of the ear is sometimes shown after removal of these masses, in which case it is prudent to shield it by wearing for a short time a little cotton-wool in the meatus.

Relief is at once given these patients when the mass escapes, and their astonishment at the change is great. The feeling of fullness in the head disappears, and with it the vertigo, and frequently, too, the tinnitus. The changed expression of the countenance of the patient from dullness and depression to hopefulness and cheerfulness is unmistakable.

---

HARD TIMES FOR PROFESSIONAL MEN IN ENGLAND.—From every part of the United Kingdom we hear news of stagnation in trade, and, consequent upon this, lower wages and hardship pressing upon every class of society. The landlord cannot get his rents because the farmer is unable to make his land pay; the manufacturer cannot run his mills except at a loss, and in many cases either stops his machinery or runs half time, thus directly affecting his operatives; the professional man, who lives by the other money-earning classes, cannot obtain a settlement of his accounts, and we have a long chain of cause and effect springing from bad trade. When wages were high, when trade was good, and the country in the highest state of prosperity, the doctor's bill was always the last to be paid, and the long period of credit given by the majority of practitioners fostered the idea that medical men, as they had such large profits, could afford to wait for their money. Now that trade is bad, medical men are suffering very severely, and in many towns there is a positive difficulty amongst practitioners who are doing large practices, to make even ends meet. Patients are as numerous as ever, population goes on increasing, despite of financial difficulties, but money is scarce.

Who is to blame? The answer is a difficult one. The operative might have saved more from the high wages he received in better times, and medical practitioners would also have been better off, if they had insisted on shorter terms of payment, and not given such long credit. We are waiting for the lifting of the cloud, also hoping that hard times will teach a salutary lesson.—*Medical Press and Circular.*

